# The index of prediction accuracy: an intuitive measure useful for evaluating risk prediction models

**DOI:** 10.1186/s41512-018-0029-2

**Published:** 2018-05-04

**Authors:** Michael W. Kattan, Thomas A. Gerds

**Affiliations:** 1 Department of Quantitative Health Sciences, Lerner Research Institute, Cleveland Clinic, 9500 Euclid Avenue/JJN3-01, Cleveland, OH 44195 USA; 2 Department of Public Health, Section of Biostatistics, University of Copenhagen, N.J. Fjords Alle 12, 4 th 1957 Frederiksberg, Øster Farimagsgade 5, 1014 Copenhagen, Denmark

**Keywords:** Prediction, Accuracy, Brier score

## Abstract

**Background:**

Many measures of prediction accuracy have been developed. However, the most popular ones in typical medical outcome prediction settings require additional investigation of calibration.

**Methods:**

We show how rescaling the Brier score produces a measure that combines discrimination and calibration in one value and improves interpretability by adjusting for a benchmark model. We have called this measure the index of prediction accuracy (IPA). The IPA permits a common interpretation across binary, time to event, and competing risk outcomes. We illustrate this measure using example datasets.

**Results:**

The IPA is simple to compute, and example code is provided. The values of the IPA appear very interpretable.

**Conclusions:**

IPA should be a prominent measure reported in studies of medical prediction model performance. However, IPA is only a measure of average performance and, by default, does not measure the utility of a medical decision.

**Electronic supplementary material:**

The online version of this article (10.1186/s41512-018-0029-2) contains supplementary material, which is available to authorized users.

## Background

A critical question for a statistical prediction model is how accurately it predicts. This assessment must be done in a manner that informs the potential end user of the model. When the outcome is a medical one, the key end user is the clinician.

Clinicians, appropriately, demand interpretability when examining measures of clinical statistical prediction models. The reason for this is that the decision to use a statistical prediction model with a patient is up to the clinician, so he or she needs to understand the performance relative to other counseling and decision making options. Presumably, for reasons related to interpretability, the concordance statistics, including Harrell’s c-index [1, 2] and the area under the (time-dependent) ROC curve [3–5], have found dramatic popularity. Certainly, they are quite intuitive and relatively easy to interpret, at least for pairs of subjects. However, it is never of interest to counsel a pair of patients, so this ease of interpretation is of little value. Moreover, these measures reflect only discrimination, and not calibration, which is both a feature and a drawback. For the modeler, it is useful to have a measure that isolates discrimination; however, this isolation requires that calibration also be assessed for a comprehensive performance analysis [6]. Also, the concordance statistics do not distinguish between a useless model and a harmful model. By that, we contrast a model that predicts the actual event proportion for all subjects (useless for discrimination) from a model that predicts a random number between 0 and 1 (also useless but also harmful because it randomly motivates action) from a model that randomly predicts 0 and 1 s (harmful, because it more strongly motivates action). For example, a model that constantly predicts 0.5, depending on the context of course, might not trigger much response to action from the user. This model will simply always predict a middle of the road outcome for all patients, which does not feel very actionable. However, a prediction of 0 or 1 will spur the user into action, potentially to deny or treat the patient with certainty. All three of these models will have a concordance statistic of 0.5. In our view, it makes sense for the harmful model (incorrectly predicting certainty) to have a worse score than a useless model (always predicting prevalence), which in turn has a worse score relative to a model that predicts with some level of predictive ability.

Also important when choosing a measure of prediction accuracy is attention to the time horizon of the prediction. Unfortunately, a drawback of Harrell’s c-index for the time to event and competing risk settings is that the measure does not provide a value specific to the time horizon of prediction (e.g., a 3-year risk) [7]. In other words, the performance calculations are not adapted to reflect the time point of the outcome being predicted; only the predicted probabilities might change when, for example, assessing a 3-year prediction vs. a 5-year prediction. One would not be able to detect whether a model’s predictions better correspond with outcome at 3 years vs at 5 years; the performance metric ought to be specific to the time horizon of prediction.

**Fig. 1:**
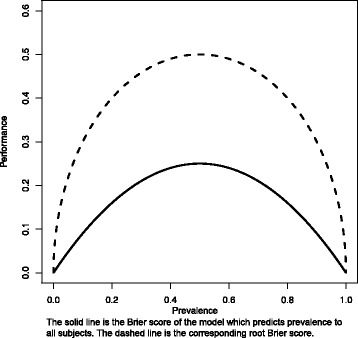
Performance metric as a function of event prevalence. Legend: the solid line is the Brier score of the model which predicts prevalence to all subjects. The dashed line is the corresponding root Brier score

Also somewhat commonly reported is the Brier score [8]. Importantly, it overcomes the concordance index limitations as it does distinguish useless from harmful models [9]. This is because the Brier score reflects both calibration and discrimination. Even better, the Brier score can support the conclusions from a graphical calibration curve which (1) can confuse the eye and (2) its interpretation depends on the person who reads the curve. In addition, the Brier score is estimated specifically for a time-specified horizon. Thus, the Brier score has many advantages over the concordance index. However, the Brier score is somewhat less interpretable by clinicians because it requires that the performance of a model is compared to the performance of the best of the useless models. The latter is a model that predicts the overall event risk for all subjects, and its Brier score (lower is better) depends on the overall event risk (see Fig. 1). This data-dependent reference value complicates interpretation of the Brier score; in absolute value, it is not always easy to know if a model is performing better than useless, because the “useless” benchmark depends on the overall event risk.

The purpose of this paper is to popularize a measure which scales the Brier score with the benchmark value, the index of prediction accuracy (IPA), and illustrate how it can be adapted to multiple settings when examining the performance of a statistical prediction model applied to a validation dataset. Specifically, we show how IPA can be used in binary, time to event outcomes, and with or without competing risks with the same interpretation (though not across the different outcomes). IPA thus reflects both discrimination and calibration, distinguishes useless from harmful models, and is specific to the prediction horizon when the outcome is time until event (with or without competing risks). Moreover, IPA quantifies the performance of a risk prediction model on a scale somewhat interpretable by a clinical audience.

## Methods

To obtain the IPA measure, we rescale the Brier score in all three settings. The value of IPA is obtained as 1 − (model Brier score/null model Brier score), where the null model contains no predictors. In the binary outcome setting, the null model simply predicts the overall prevalence of the outcome in the validation dataset. However, for the time to event and competing risk settings, censoring needs to be accommodated, and as such, a time horizon must be chosen, and the null model needs to be estimated with the Kaplan-Meier [10] (no competing risks) or the Aalen-Johansen method [11] (with competing risks). Furthermore, in the case of right-censored observations, the Brier score is estimated with the use of inverse probability of censored weighting estimation [8, 12]. After using this technique to calculate the model and null model Brier scores, the IPA measure is constructed as above. This yields an IPA measure for all 3 settings (binary, time to event, and competing risks) with common interpretation: 100% is a perfect model, ≤ 0 is a useless model, higher is better, and harmful models have IPA < 0.

**Fig. 2:**
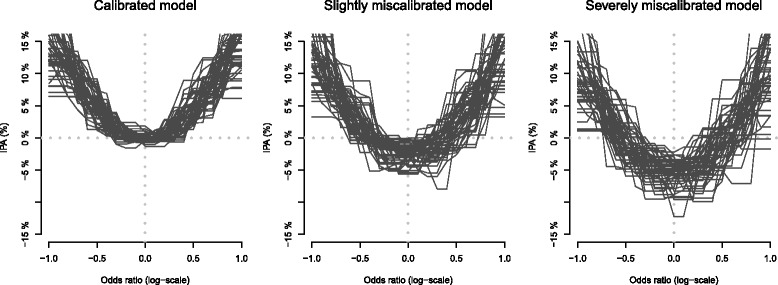
Illustration of IPA as a function of discrimination, without and with miscalibration

We first performed a series of simulations to illustrate how the IPA measure captures both calibration and discrimination, also comparing with AUC. For this purpose, we used uncensored binary outcome data and a single continuous predictor variable with standard normal distribution. We simulate outcome data from a logistic regression model with odds ratio values varying on the logarithmic scale between − 1 and 1. For each value of the odds ratio, we generate three learning datasets (*n* = 1000) which differ with respect to their marginal prevalence of the outcome (40, 25, and 10%). We then calculate IPA and AUC using an independent validation dataset (*n* = 200, marginal prevalence = 40% as in the first learning dataset) for a calibrated logistic regression model (fitted in the first learning dataset) and miscalibrated logistic regression models (fitted in the second and third learning datasets). The fitting and testing was performed 50 times. In Fig. 2, we show the results which nicely illustrate that the perfectly calibrated model falls to an IPA of 0% as the model loses all discrimination (i.e., the odds ratio reaches 0). The miscalibrated models, which also have no discrimination for an odds ratio near 0, have an IPA < 0%. A strength of the IPA measure is that it provides a worse value for the miscalibrated model even though both models have no discrimination when the odds ratio reaches zero. Moreover, the IPA measure worsens with increasing miscalibration.

### Data for illustration

**Fig. 3:**
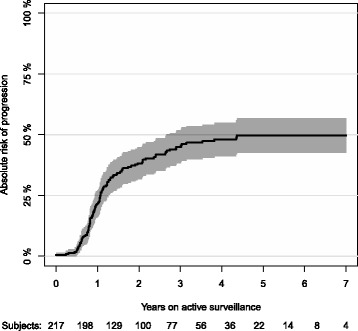
Absolute risk of progression accounting for non-cancer death as a competing risk

We randomly split the data of a prostate cancer active surveillance study [13] into a learning set (*N* = 137) and a validation set (*n* = 80). We do this for the purpose of illustration and note that the results depend considerably on the random seed used for splitting data. The risk of progression in this dataset appears in Fig. 3.

### IPA for the binary outcome setting

To illustrate the binary outcome setting, we analyze the 1-year progression status of the active surveillance study [9]. We have complete 1-year follow-up of the 217 (137 + 80) patients for whom we also have a minimum of three PSA measurements, one rebiopsy, and complete diagnostic information. Within the first year, 47 patients progressed and 3 died. We fit a logistic regression model including cT stage, diagnostic GS, and ERF status as categorical variables and age, percentage of positive biopsies (PPB), PSA density, and maximum tumor involvement as continuous variables.

#### Results

The Brier scores of the full logistic regression model (7 variables) and null model (0 variables) were 16.2 and 17.4%, respectively. The null model is obtained when all of the 7 variables are dropped from the model. The IPA values are contained in Table 1. The column IPA gain (%) shows the difference in IPA between the full model and the reduced model where the current variable is dropped. The largest drop in IPA was observed for the ERG status (6.3%). Note that a negative value of IPA gain for a variable indicates that the model without this variable outperforms the full model.

**Table 1:** Results for the binary outcome setting. Brier (full model) 16.2 and Brier (null model) 17.4

Variable	Units	Odds ratio	95% CI	*p* value	IPA (%)
Full model					7.28
					Loss in IPA (%) compared to full model
Age	5 years	0.87	[0.46;1.66]	0.68	0.28
PSA density	Twofold	1.40	[0.77;2.55]	0.26	2.69
Percentage of positive biopsies	5 points	1.10	[0.85;1.43]	0.47	0.62
Maximum tumor involvement	Twofold	1.59	[1.01;2.50]	0.05	3.11
cT stage	cT1	Ref			− 0.08
	cT2	1.12	[0.28;4.43]	0.87	
Diagnostic GS	GNA	Ref			− 2.39
	3 and 3	0.61	[0.13;2.81]	0.53	
	3 and 4	1.29	[0.18;9.33]	0.80	
ERG status	Negative	Ref			6.29
	Positive	3.17	[1.22;8.26]	0.02	

### IPA for the survival outcome setting

To illustrate the survival outcome setting, we analyze the 3-year chance of progression-free survival in the active surveillance study. We have up to 7.7 years of follow-up of the same 217 (137 + 80) patients already used in the binary setting. In this study, the median (IQR) potential follow-up time was 4.81 years (3.94; 5.37). For the purpose of illustration, we choose a 3-year prediction horizon. Within 3 years, 127 patients had the event (progressed or died) and 69 were alive and event-free. The remaining 21 patients were lost to follow-up before 3 years; their status at 3 years is thus unknown to us. We fit a Cox regression model including cT stage, diagnostic GS, and ERG status as categorical variables and age, percentage of positive biopsies (PPB), PSA density, and maximum tumor involvement as continuous variables.

#### Results

For the 3-year prediction horizon, the Brier scores of the full Cox regression model (7 variables) and null model (0 variables) were 21.4 and 23.6%, respectively. This corresponds to an IPA value of 9.2%. Table 2 shows the change in IPA for the 3-year prediction horizon when each of the 7 variables was dropped from the model. The interpretation of the column IPA gain is the same as in the binary setting. The largest drop (see Table 2) was observed for the ERG status (7.6%). As in the binary setting, negative IPA gain values mean that dropping the variable improves the prediction accuracy compared to the full model.

**Table 2:** Results for the survival outcome setting. Brier (full model) 21.4 and Brier (null model) 23.6

Variable	Units	Odds ratio	95% CI	*p* value	IPA (%) at 3 years
Full model					9.21
					Loss in IPA (%) compared to full model
Age	5 years	1.18	[0.87;1.61]	0.27	− 0.57
PSA density	Twofold	1.06	[0.84;1.35]	0.61	1.26
Percentage of positive biopsies	5 points	1.08	[0.95;1.23]	0.24	3.47
Maximum tumor involvement	Twofold	1.20	[0.98;1.47]	0.08	− 4.34
cT stage	cT1	Ref			− 2.49
	cT2	1.68	[0.91;3.13]	0.10	
Diagnostic GS	GNA	Ref			3.17
	3 and 3	0.65	[0.33;1.27]	0.21	
	3 and 4	1.15	[0.46;2.90]	0.77	
ERG status	Negative	Ref			7.60
	Positive	1.48	[0.97;2.25]	0.07	

### IPA for the competing risk setting

To illustrate the competing risk setting, we analyze the risk of progression accounting for non-cancer death as a competing risk in the active surveillance study. We use the Aalen-Johansen method [11] to estimate the absolute risk of progression in the follow-up period (see Fig. 3).

To illustrate that the prediction horizon can be chosen by the user, we now set it at 4 years. Within 4 years on active surveillance, 100 patients had progressed, 42 died, and 38 were alive and event-free. The remaining 37 patients were lost to follow-up before 4 years; their status at 4 years is thus unknown to us. Overall, the 4-year absolute risk of progression is estimated as 48.1% (95% CI 41.2–55.0) and the 4-year absolute risk of non-cancer deaths as 22.5% (95%CI 16.4–28.5). The null model predicts a risk of 48.1% to all patients no matter their risk profile.

We fit a cause-specific Cox regression model for the hazard rate of progression including cT stage, diagnostic GS, and ERG status as categorical variables and age, percentage of positive biopsies (PPB), PSA density, and maximum tumor involvement as continuous variables. We also fit a cause-specific Cox regression model for the hazard rate of non-cancer death (without progression) including only age as a predictor variable. Based on these models and using the formula of Benichou and Gail [14], we predict the patient individual absolute risk of progression. As an alternative, we also fit a Fine and Gray [15] regression model including cT stage, diagnostic GS, and ERF status as categorical variables and age, percentage of positive biopsies (PPB), PSA density, and maximum tumor involvement as continuous variables.

#### Results

**Fig. 4:**
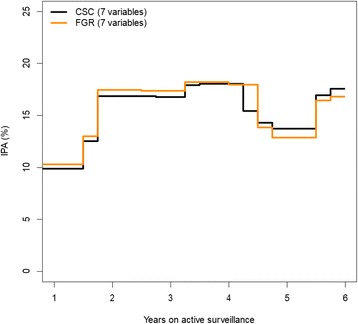
Illustration for the effect on IPA from changing the prediction horizon

We focus our attention on the risk of progression. For the 4-year prediction horizon, the Brier scores of the full cause-specific Cox regression model (7 variables for progression and 1 variable for non-cancer death) and null model (0 variables) were 20.4 and 25.0%, respectively. This corresponds to an IPA value of 18.3%. Table 3 shows the change in IPA for the 4-year prediction horizon when each of the 7 variables was dropped from the two cause-specific Cox (CSC) regression models (similar results are obtained with the Fine and Gray regression model, data not shown). The interpretation of the column IPA gain is the same as in the binary setting. The largest drop (see Table 3) was observed for the ERG variable (9.0%). Also here, a negative IPA gain means that dropping the variable improves IPA. Figure 4 shows for the full CSC model (7 variables for progression hazard and 1 variable for hazard of progression-free death) and the Fine and Gray model (7 variables for subdistribution hazard of progression) how IPA varies with an increasing prediction horizon.

**Table 3:** Results for the competing risk outcome setting. Brier (full model) 20.4 and Brier (null model) 25.0

Variable	Units	Odds ratio	95% CI	*p* value	IPA (%) at 4 years
Full model					18.26
					Loss in IPA (%) compared to full model
Age	5 years	1.00	[0.69;1.46]	0.981	− 0.20
PSA density	Twofold	1.03	[0.76;1.39]	0.870	0.45
Percentage of positive biopsies	5 points	1.04	[0.90;1.21]	0.605	1.85
Maximum tumor involvement	Twofold	1.26	[0.98;1.62]	0.070	− 1.53
cT stage	cT1	Ref			− 2.40
	cT2	1.90	[0.93;3.89]	0.077	
Diagnostic GS	GNA	Ref			6.20
	3 and 3	0.66	[0.27;1.58]	0.347	
	3 and 4	1.51	[0.51;4.52]	0.458	
ERG status	Negative	Ref			9.01
	Positive	2.23	[1.30;3.83]	0.004

## Software

The R package “riskRegression” contains all the necessary code for running the calculations. Example syntax is provided in Additional file 1.

## Discussion

We have illustrated how the Brier score can be adapted to a single value measure (IPA) with friendly interpretation. This IPA measure overcomes many of the limitations of the widely popular concordance index and is more interpretable than the Brier score. The idea to use Brier score to construct our measure is not new [11, 12]. Our contribution is to point out that the same definition holds for all three outcome measures and our freely available software (see Additional file 1). This measure should be useful as a supplement to report when evaluating predicted probabilities in the most popular medical outcome settings of binary, time to event, or competing risks. Thus, our IPA measure has applicability when testing the performance of models constructed using logistic regression, Cox regression, competing risk regression, or absolute risk regression.

IPA has important limitations. It is a measure of average performance and may not reflect improvements that affect a small subset of the population. IPA depends on the marginal outcome probability and, therefore, cannot easily be compared across different studies or populations. IPA does not, by default, incorporate the medical decision and thus does not measure the clinical utility of the model. For example, reference [9] shows how one can incorporate the cost-benefit ratio into the Brier score and hence into IPA. However, in most applications, it is difficult to determine a single value which represents the cost-benefit ratio of all patients. This is also reflected by decision curve analysis [16] where the results depend on the patients’ personal threshold of cost and benefit associated with the clinical decision.

Indeed, it is useful to think of the utilization of prediction models for decision making purposes to be a two-step process. In the first step, the focus is on getting the best model, as measured by calibration and discrimination, for use in predicting the risk of the event for the current patient. In the second step, a medical decision is made based on the predicted risk. The decision will usually depend on additional information, such as the judgment of a clinician or patient preferences. Moreover, competing risks may need to be considered. Because of this complexity of preferences and multiple endpoints, two patients may have different clinical decisions even if they have received the exact same predicted risk of a particular event. In a setting where it is possible to quantify the benefit/regret of the consequences of the second step, decision curve analysis combines the two steps by advocating the model that achieves the highest benefit. Similarly, it is possible to integrate differential costs of the decision into the Brier score [9, 17]. However, complete information regarding the second step is often not available on a patient-specific level during the development phase of the model as this would require knowledge regarding how each patient would have decided when the risk was 9% (model 1) instead of 12% (model 2). IPA can be used in settings where decision making is a two-step process, for the first step, to find a calibrated model with high discrimination. The evaluation of the combination of the two steps where the model does not predict the risk but directly shows the medical decision is beyond the scope of this paper.

## Conclusions

**Fig. 5:**
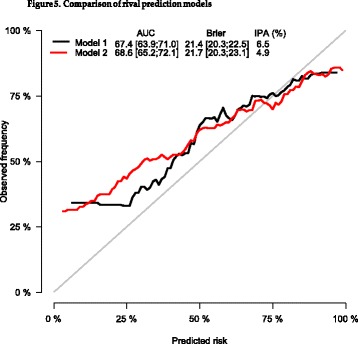
Comparison of rival prediction models

Arguably, this IPA measure should be an initial performance metric when evaluating statistical prediction models in these common biomedical settings (binary, time to event, and competing risk). By reflecting calibration, and not just discrimination, IPA may be more likely to identify the preferred model when rivals are competing. For example, see two calibration curves in Fig. 5. Most would presumably say that the black curve is better calibrated than the red curve. Interestingly, the red model has better discrimination, but a worse IPA value, than the black model. Thus, the concordance index leads to the selection of the wrong model, while the IPA metric selects the better calibrated model.

## Additional file

Additional file 1: Example syntax. (PDF 91 kb)
