# Supplementary material for: The index of prediction accuracy: an intuitive measure useful for evaluating risk prediction models
Source: Diagn Progn Res. 2018 May 4;2:7. doi: 10.1186/s41512-018-0029-2 (PMC6460739; doi:10.1186/s41512-018-0029-2)
Supplement: Supplementary file 1 — Example syntax. (PDF 91 kb) [file 41512_2018_29_MOESM1_ESM.pdf]

Additional file 1

```
astrain <- get(load("path/to/astrain.rda"))
astest <- get(load("path/to/astest.rda"))

# binary based on logistic regression
library(riskRegression)
library(survival)
lrfit <- glm(Y1~ct1+diaggs+erg.status+age5+lpsaden+ppb5+lmax,
             data=astrain,
             family="binomial")
IPA(lrfit,newdata=astest)

# survival based on Cox model
coxfit <- coxph(Surv(asprogtime,asprog!=0)~ct1+diaggs+erg.status+age5+lpsaden+ppb5+lmax,
                data=astrain,
                x=1)
IPA(coxfit,newdata=astest,times=3)

# competing risks based on cause-specific Cox models
cscfit <- CSC(list(Hist(asprogtime,asprog)~ct1+diaggs+erg.status+age5+lpsaden+ppb5+lmax,
                   Hist(asprogtime,asprog)~age5),
              cause="progression",
              data=as)
IPA(cscfit,cause="progression",newdata=astest,times=3))

# rsquared for a given risk prediction
set.seed(18)
p <- runif(NROW(astest),0,1)

#binary
IPA(p,formula=Y1~1,newdata=astest)
#survival
IPA(p,formula=Hist(asprogtime,asprog!=0)~1,newdata=astest,times=3)
#comprisk
IPA(p,cause="progression",formula=Hist(asprogtime,asprog)~1,newdata=astest,times=3)
```
